# The American Medical Library

**Published:** 1839-10

**Authors:** 


ART. XI.—*The American Medical Library and Intelligencer.* Published Semi-Monthly. Second year: from April 1838 to April 1839. Edited by ROBLEY DUNGLISON, M.D., Professor of the Institutes of Medicine in Jefferson Medical College, &c.—*Philadelphia*, 1838-1839. 8vo.

WE noticed this publication on its first appearance, and refer for its general plan to our Fourth Vol., p. 495. It has proceeded since that time with uninterrupted regularity, and every part bears evidence of the learning, industry, and sound discretion of the editor. Although, as we said on the former occasion, this plan of wholesale republication cannot fail to be injurious to the pecuniary interests of the authors and proprietors of medical works in this country, there is still some compensation afforded to those who write for fame or for the good of their fellow-creatures, in the ready and speedy diffusion of their works, by means of this "Library," throughout the vast country in which it circulates. The following are the works reprinted during the last twelve months, and which (including *The Intelligencer*, forming a large volume in itself,) are sold for ten dollars—about one sixth of the cost of the separate volumes as imported!—Dr. Kramer, on Diseases of the Ear; Dr. Hamilton's Practical Observations on Midwifery; Mr. Syme, on Diseases of the Rectum; Dr. Osborne, on Dropsical Diseases; Dr. Green, on Diseases of the Skin; Mr. Coulson, on Diseases of the Bladder; Mr. Liston's Practical Surgery (with Notes by Dr. Norris); Mr. Morgan, on Inflammation; Dr. Granville, on Counter-Irritation; Dr. Hodgkin, on the Morbid Anatomy of Membranes; Mr. Ryland, on Diseases of the Larynx and Trachea; Dr. Rowland, on Neuralgia; Dr. Dunglison, on the condition of the Insane Poor; Dr. Churchill, on the Diseases of Females; Prof. Lallemand, on Seminal Discharges (translated); Dr. Clendinning's Croonian Lectures; Sir A. Cooper, on Spermatocele.

Henceforth English medical authors will not have to complain that they are not read by the profession in America, at least. We hope that this ready, easily-accessible, and never-failing supply from a foreign source will not tend to dry up or to let stagnate the rich well-spring of native talent in America. If such should prove to be, in any respect, the case, we think, even at the moderate cost of "ten dollars a year," our Transatlantic brethren would—in the language of their greatest citizen—"pay much too dear for their whistle."

